# Unmasking the Masquerade: Fine-Needle Aspiration Diagnosis of Dedifferentiated Liposarcoma Clinically Mimicking Lymphoma

**DOI:** 10.7759/cureus.55759

**Published:** 2024-03-07

**Authors:** Prerna Chadha, Raghav Kapoor, Poojan Agarwal, Sunil Pasricha, Anurag Mehta

**Affiliations:** 1 Pathology, Rajiv Gandhi Cancer Institute and Research Center, New Delhi, IND; 2 Pathology, Jawaharlal Nehru Medical College (KLE), Belagavi, IND; 3 Pathology and Laboratory Medicine, Rajiv Gandhi Cancer Institute and Research Center, New Delhi, IND; 4 Cytopathology, Sir Gangaram Hospital, New Delhi, IND; 5 Research and Pathology, Rajiv Gandhi Cancer Institute and Research Center, New Delhi, IND

**Keywords:** liposarcoma, morphology, ihc, cytology, lymphoma, dedifferentitated liposarcoma

## Abstract

A preoperative diagnosis of dedifferentiated liposarcomas (DDLPS) on fine-needle aspiration cytology (FNAC) is rare with scarce indexed literature. Herein, we describe a case of DDLPS diagnosed on fine needle aspiration which was presumed to be a lymphoma clinically and radiologically.

## Introduction

Soft tissue sarcomas can arise at a range of anatomical sites such as the extremities, retroperitoneum, head and neck, and subcutaneous fat [1]. Liposarcoma is the most prevalent type of soft tissue sarcoma, making up approximately 20%-35% of all soft tissue sarcomas [2]. According to the most recent World Health Organization classification, liposarcomas are now categorized into five distinct groups: Well-differentiated or atypical lipomatous tumor, dedifferentiated liposarcomas (DDLPS), myxoid, pleomorphic and myxoid pleomorphic. Well-differentiated liposarcoma includes the adipocytic, sclerosing, and inflammatory subtypes [3]. A preoperative diagnosis of DDLPS on fine-needle aspiration cytology (FNAC) is rare with only a few cases described in indexed literature. Herein, we describe a case of DDLPS which was presumed to be a lymphoma clinically and radiologically. The diagnosis was made on the basis of cytology and the findings of this rare entity are reemphasized with a short review of the literature.

## Case presentation

The patient was a 65-year-old hypertensive male who presented with complaints of left flank pain (renal colic) and weight loss of three months duration. On investigation, he was incidentally detected with a porta hepatic mass on Ultrasonography. Contrast-enhanced CT scan (CECT) whole abdomen was done which revealed multiple hypo-enhancing enlarged and conglomerate nodal masses at porta hepatic, peri-pancreatic, pre & para-aortic regions. Similar lesions were also seen involving the mesentery along the transverse colon on the left side and along the left renal capsule. A provisional diagnosis of lymphoma was given in radiology. Eventually, Positron emission tomography (PET-CT) was performed which showed metabolically active heterogeneously enhancing confluent soft tissue mass lesion measuring 61 x 58mm (SUV max. 5.3) involving the portal/periportal, portocaval, gastrohepatic, celiac axis and peripancreatic, nodal stations. Metabolically active discrete similar lymph nodal mass was also seen in the para-aortic region (36 x 35mm, SUV max. 8.9) and left perirenal region (44 x 42mm, SUV max. 11.2). The serum LDH levels were 308 units/L (normal 105-233). The patient underwent Endoscopic ultrasound (EUS) guided FNA and biopsy (FNB) which was reported as a myxoid spindle cell sarcoma, possibly low-grade fibromyxoid sarcoma at an outside hospital. The patient was referred to our tertiary cancer care hospital for a second opinion and the slides and paraffin-embedded block were reviewed.

Methods

**Figure 1:**
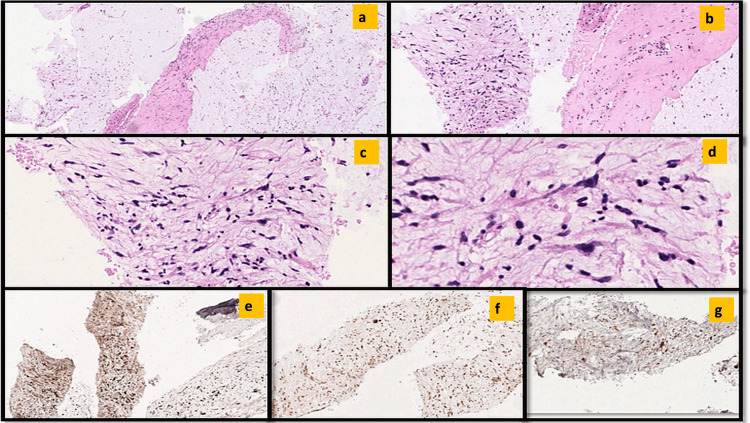
Cytopathological images of EUS-FNA from the periportal mass (a-d) Giemsa-stained slides showing intermediate to large sized tissue fragments with anastomosing vessels embedded within a myxoid matrix. Arrows highlight the singly dispersed population of tumor cells. (e-g) Tumor cells at the periphery showing significant nuclear atypia.

Informed consent was given by the patient. EUS-FNA from the periportal mass showed a cellular aspirate having intermediate to large-sized tissue fragments with anastomosing vessels embedded in a myxoid matrix (Figures 1a-1f). These tissue fragments were composed of atypical plump spindle-shaped cells having hyperchromatic nuclei and inconspicuous nucleoli. Moderate nuclear pleomorphism was noted. A singly dispersed population of tumor cells with bipolar cytoplasmic processes exhibiting marked nuclear pleomorphism with the presence of prominent nucleoli was also noted (Figures 1b, 1f). Interspersed lymphoid cells and neutrophils were seen within the tissue fragments at places. Apoptosis was noted. The background showed hemorrhage. No definite lipoblast was seen.

**Figure 2:**
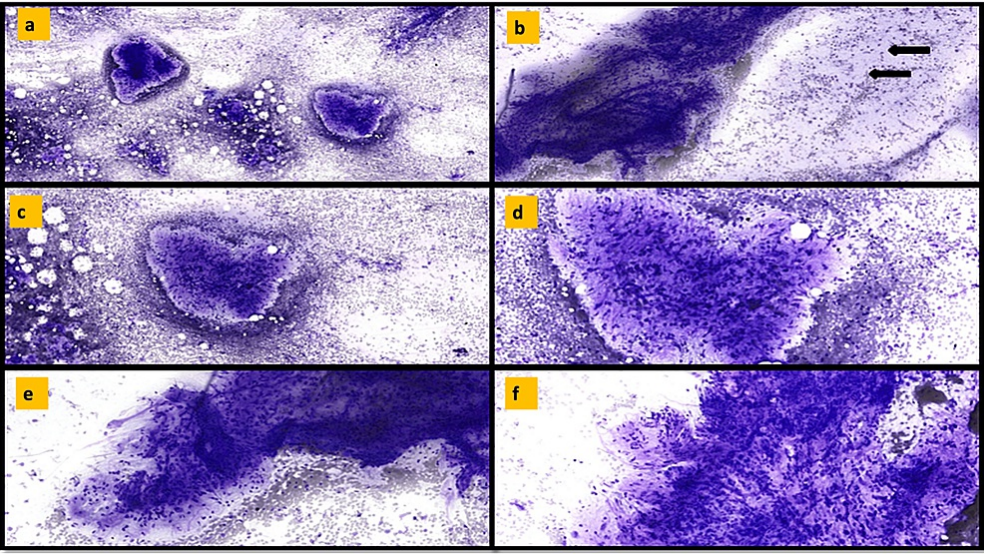
EUS-FNAB images from the periportal mass (a-c) Histopathological images showing a spindle cell tumor with hypercellular and hyocellular areas and a myxoid background. (d) Markedly pleomorphic cells. (e-g) Immunohistochemical positivity for CDK4, MDM2, and p16, respectively.

FNB comprised thin cores of a spindle cell tumor with a myxoid background and thin-walled vasculature interspersed throughout the tumor. Moderate nuclear pleomorphism was seen. No adipocytic differentiation was identified (Figures 2a-2d). An occasional tiny strip of benign gut epithelium was also included. On immunohistochemistry (IHC) the tumor cells expressed CDK4, MDM2, and P16 (Figures 2e-2g) while being negative for CK, S100, SMA, and CD117.

Based on EUS-FNA, FNB, and IHC, a final diagnosis of dedifferentiated liposarcoma with minimum Fédération Nationale des Centres de Lutte Contre le Cancer (FNCLCC) Grade 2 was rendered.

## Discussion

DDLPS represents well-differentiated liposarcoma which commonly undergoes an abrupt transition to a non-lipogenic sarcoma with a variable histomorphological grade. This transition may occur either de novo or at the time of recurrence [3]. The tumor most commonly occurs in the fifth to seventh decades of life with a predilection for retroperitoneal location. DDLPS is associated with a high incidence of local recurrence, a metastatic rate of 15%, and an approximately 30% disease-related mortality rate [4]. Hence, it is imperative to recognize and correctly diagnose the same preoperatively. Owing to the misleading appearance of these malignant tumors clinically as well as radiologically, it is essential to perform comprehensive preoperative investigations. FNAC plays a vital role at this point. It is easy to perform, cost-effective and most of the time yields an accurate diagnosis [5]. In our case too, the clinical opinion was that of a lymphoma which differs starkly from a liposarcoma in terms of prognosis and treatment strategy.

Diagnosing sarcoma through FNA can be challenging due to similar cytomorphology seen in various soft tissue lesions, especially spindle cell lesions [6]. FNA is readily used in documenting metastasis and recurrence of mesenchymal neoplasms, however, reports of primary diagnosis on cytology are scarce [7]. Sarcomas usually show hemorrhage, necrosis, dense fibrosis, or matrix material which might obscure the morphology of viable tumor cells. Diagnosis of DDLPS can be difficult and complicated further because of the scant cellularity of the aspirates due to retroperitoneal location and occurrence of a wide variety of morphological growth patterns including, spindle cell, pleomorphic, inflammatory, giant cell, round cell, or even meningothelial-like patterns [8]. As a consequence of such impediments, there are only a few cytologic descriptions of DDLPS in indexed literature so far [9]. Overlapping cytomorphological features such as the presence of a myxoid background and rich vasculature, as seen in other soft tissue neoplasms such as myxofibrosarcomas, low-grade fibromyxoid sarcoma or myxoid liposarcomas may further act as a potential trap for the cytopathologist as occurred in this patient. In such cases, simultaneous cell block along with judicious use of ancillary testing such as IHC or molecular testing can aid in obtaining a correct and timely diagnosis [9,10].

Dedifferentiated liposarcomas arise from their well-differentiated counterparts and thus share certain cytogenetic features like amplification of MDM2 and CDK4 genes. The use of IHC to assess CDK4, MDM2, and p16 expression as part of the diagnostic armamentarium plays a crucial role in distinguishing well-differentiated and dedifferentiated liposarcomas from other adipocytic neoplasms. Detection of MDM2 not only assists in the diagnosis but also helps in determining the treatment strategy in view of the availability of targeted therapy such as MDM2 inhibitors which is especially important for metastatic or non-resectable tumors.

## Conclusions

Dedifferentiated liposarcomas are high-grade sarcomas and the diagnosis is determined by the demonstration of a non-lipogenic sarcoma with or without a well-differentiated liposarcomatous component. The cytological examination is beneficial and provides an accurate diagnosis if sampling is adequate and can also provide material for molecular or ancillary testing. A multidisciplinary team approach involving the surgeon, radiologist, pathologist, and oncologist should be present for the accurate diagnosis of this neoplastic lesion which can sometimes pose a diagnostic challenge for the cytopathologist.
